# Effect of DEET-multiple exposures on behavior and life history traits in the malaria mosquito *Anopheles gambiae* (*s.s.*)

**DOI:** 10.1186/s13071-018-3024-0

**Published:** 2018-07-25

**Authors:** Margaux Mulatier, Ludovic Phamien Ahoua Alou, Fabrice Chandre, Cédric Pennetier, Laurent Dormont, Anna Cohuet

**Affiliations:** 1 MIVEGEC, IRD, CNRS, University of Montpellier, Montpellier, France; 2 CEFE, University Paul Valéry Montpellier 3, CNRS, University of Montpellier, EPHE, IRD, Montpellier, France; 3 Institut Pierre Richet, Bouaké, Côte d’Ivoire

**Keywords:** DEET, Multiple-exposures, *An. gambiae*, Experience, Fitness

## Abstract

**Background:**

Vector-borne diseases are major public health concerns, and their control is threatened by the spread of insecticide resistance in vector populations. In this context, the use of repellents is an alternative approach to limit vector-host interactions. However, prior exposure to repellents is suspected to affect mosquito behavior at the subsequent exposure, possibly reducing the efficacy of the compound. Despite this, the effect of mosquito experience on repellent efficacy remains poorly documented. In the present study, we tested whether a first blood meal successfully obtained upon a DEET-treated net would affect the success at taking a second blood meal in spite of DEET in the malaria mosquito *Anopheles gambiae* (*s.s.*). The impact of DEET on mosquito life history traits after the first and the second exposure was also measured, in order to assess the long-term consequences of multiple exposures to DEET in vector insects.

**Results:**

A first blood meal obtained upon a DEET-treated net did not influence the success of *An. gambiae* females to take a second blood meal in spite of DEET. However, data showed that a prior exposure to DEET negatively affected all life history traits tested in this study related to fecundity and fertility. DEET pre-exposed females displayed a reduction in blood engorgement at the second exposure, as well as a reduction in the number of eggs laid and in the proportion of offspring that reach adult stage. Also, an increase of mosquito activity was observed during the second blood meal in DEET-pre-exposed females. Taken together, these data suggest an overall impact of DEET exposure on mosquito fitness.

**Conclusions:**

Our results did not evidence any effect of a prior exposure to DEET on its efficacy during the second exposure. However, data show a negative impact of DEET exposure on mosquito fitness. These results give insights to understand the long-term efficacy of the most used mosquito repellent, and highlight that DEET induces deleterious effects on mosquito fitness in addition to repellency, potentially increasing its efficacy for controlling vector-borne diseases.

## Background

Vector-borne diseases are major public health threats, accounting for more than 17% of all infectious diseases and responsible for about 700,000 deaths annually [1]. Mosquitoes are responsible for the transmission of a large number of vector-borne pathogens causing these diseases. For example, the deadly malaria parasites are transmitted to humans by *Anopheles* mosquitoes, and emerging arboviruses such as dengue, chikungunya or the Zika virus are vectored by *Aedes* mosquitoes. To date, vector control remains the most efficient method to fight against these diseases, and mostly relies on the use of pyrethroid insecticides. However, resistance mechanisms to insecticides have evolved and are now widespread in the targeted populations [2, 3], which threatens the efforts made in the control of vector-borne diseases. Moreover, the increased number of arboviral disease outbreaks in intertropical regions [4, 5] and the expansion of *Ae. albopictus* beyond tropical areas [6, 7] entail an urgent need for new control strategies. In this context, the use of repellents has gained increased interest for vector control and their potential is under scrutiny. Repellents have proven efficacy to inhibit the blood meal of *Anopheles* and *Aedes* mosquitoes when impregnated on nets or clothes [8–10]. Moreover, when applied together on nets with non-pyrethroid insecticides, the repellent DEET (N,N-diethyl-3-methylbenzamide) and the insecticide revealed synergistic activity [11, 12]. Interestingly, the mixture restored a knockdown effect and mortality similar to pyrethroids against multi-loci resistant mosquitoes [13, 14]. Such synergistic effect was also observed, when used against pyrethroid-resistant mosquitoes, between repellents (both DEET and IR3535) and a pyrethroid insecticide impregnated with long-lasting polymer coating [15]. All together, these studies suggest that repellents may have important potential in vector control when impregnated singly or in mixtures on fabrics. The use of repellents also appears to be a promising tool to target vector insects that are not reached by current insecticide-based methods, for instance conferring an outdoor protection [16].

DEET is estimated as the most broad-spectrum and efficient insect repellent [17]. Although its efficacy has been widely proven, it remains overlooked if DEET entails constant repellency during the whole lifespan of the targeted organism. For instance, in areas of high repellent coverage, insects may face multiple exposures to DEET during their life. This could generate modifications in the behavioral response due to the effect of experience, sensory habituation, desensitization, or more complex learning abilities [18]. Understanding how repellents efficacy may change over mosquito lifetime may have a significant impact on their use for disease control. Of particular interest for most vector-borne diseases affecting humans is that the source of infection for the vector is a previous blood meal on an infectious human. Therefore, if repellents are broadly used, infected vectors will have a high probability to have experienced a contact with a repellent. The fitness and behavioral consequences of prior exposure to repellents of vector insects then deserve attention. In this context, in mosquitoes, experimental studies analyzing the effect of multiple exposures on DEET efficacy remain marginal but are of high interest.

In *Ae. aegypti*, females pre-exposed to DEET displayed decreases in sensitivity and in electrophysiological responses to DEET at a second exposure, three hours after the first [19]. Moreover, an olfactory learning ability was evidenced in *Ae. aegypti*, when the natural aversion for DEET was rendered ineffective 24 hours after associative learning with a blood source reward [20]. Taken together, these results suggest that mosquitoes can modulate their behavior on the basis of previous events and that prior exposure to DEET may decrease its repellency to mosquitoes, at least within 24 hours following the first exposure. However, under natural conditions, most mosquitoes seek a blood meal every two to four days [21]. To our knowledge, the effects of prior exposure to repellents considering a biologically relevant period between the two blood meals have never been characterized. Additionally, besides olfactory repellency, DEET is also an irritant by contact for mosquitoes [22]. This could induce aversion [23], increasing avoidance behavior over experience with the repellent [18]. Previous exposure to DEET may thus generate contrasted effects and exploring the behavioral outcome is needed to decipher the potential of repellents in vector control strategies.

In the present study, we examined in *An. gambiae* (*s.s.*) the effects of a blood meal successfully obtained upon a DEET-treated net on the success of taking a second blood meal in spite of DEET presence using a biologically relevant time period between the two blood meals (i.e. 3–4 days). We also measured the impact of DEET exposure on life history traits after the first and the second exposure to provide a comprehensive picture of epidemiological consequences of multiple exposures to DEET in vector insects.

## Methods

### Mosquito colony

Mosquitoes were reared and tested thanks to the technical/research platform dedicated to vectors at the Institut de Recherche pour le Développement (IRD) Centre, Montpellier, France. Experiments were performed using *An. gambiae* (*s.s.*) females that had the possibility to mate and have never had access to blood-feeding. As pyrethroid resistance is already widespread throughout western and central Africa [24] and is suspected to affect DEET efficacy because of observed behavioral interactions [25], experiments were carried out on pyrethroid-resistant mosquitoes. They belong to the KdrKis strain, harboring the L1014F homozygote mutation (*kdr-west* allele) in the gene coding for the voltage-gated sodium channel. They were reared at 27 °C and 80% relative humidity with a light:dark photoperiod of 14:10 h. They were fed during the larval stage with ground TetraMin (Tetra, Melle, Germany) and in their adult stage with a 10% honey solution.

### Selection of DEET concentration and first exposure

Mosquito blood-feeding assays were carried out using 7–11 days old female mosquitoes that were starved for one day. They were distributed in paper cups (height: 10 cm, diameter: 7 cm) in groups of 25 individuals. Cups were covered with mosquito mesh and placed under glass feeders that were sealed on one end with a parafilm membrane allowing mosquitoes to feed through it. Feeders were filled with 500 μl of rabbit blood and kept at 37 °C using water bath circulation (Julabo Labortechnik, Seelbach, Germany).

To measure the effect of a successful blood meal taken through a DEET-impregnated mosquito net on the behavior at the second exposure, we selected a DEET concentration responsible for between 25–75% of mean inhibition compared to the control group. The chosen dose induces repellency, but also enables some females to successfully blood-feed and thus to be tested for the second exposure. Preliminary assays indicated that a dose of 3.45 g/l in ethanol, which allowed for a dose of 250 mg/m^2^ of DEET in impregnated net results in such an inhibition of feeding in our experimental setup. For each cup, a 15 × 17 cm piece of polyester net was therefore impregnated with 250 mg/m^2^ of DEET. Control nets were impregnated with the solvent. After impregnation, nets were left 1 h at room temperature to allow the solvent to evaporate. Impregnated nets were placed between the feeder and the paper cup mesh so that mosquitoes would contact the impregnated mesh when blood-feeding. After 1 h of exposure, blood-fed females were counted and maintained in the same conditions as during the rearing for a subsequent exposure. They were supplemented with 10% honey and had the opportunity to oviposit. A subset of the fed females was placed individually into 30 ml tubes for blood meal size measurement. After 48 h, they were pooled into a cage and were allowed to oviposit.

### Assessment of the biological relevance of the used concentration of DEET

To assess the biological relevance of the DEET concentration used for net impregnation, we compared the emanation of DEET from nets that were impregnated as described above and emanations over time from human skin sprayed with DEET solution at the recommended dose. The guidance for repellent testing recommends approximately 3 g/m^2^ of DEET for cutaneous application [26, 27]. Additionally, commercial formulations of DEET-based repellents suggest doses of 2 g/m^2^ for adult antivectorial protection [28]. For this study, 2 g/m^2^ was thus applied on the forearm of adult volunteers, on a 7 × 7 cm surface. Kinetic of DEET-emission by the skin was measured at 0, 2, 4 and 6 h after application using solid phase micro-extraction (SPME): the forearm of each subject was enclosed in a nonreactive bag made from polyethylene terephthalate (Nalophan; Kalle Nalo GmbH, Wursthüllen, Germany), and a SPME fiber (65 μm polydimethylsiloxane / divinylbenzene stableflex, Supelco, Sigma-Aldrich, Bellefonte, PA, USA) was introduced with a manual holder into the Nalophan bag, taking care not to touch the skin surface with the fiber. The fiber was then exposed in close proximity (2 cm) to the skin surface for 30 min. Five series of odor collections were carried out from four people. Seven 7 × 7 cm nets impregnated with 250 mg/m^2^ of DEET were also enclosed in separate Nalophan bags, and the headspace was similarly collected 1 h post-impregnation. SPME odor collections were analyzed by chromatography-mass spectrometry using a quadrupole mass spectrometer Shimadzu QP2010 Plus (Shimadzu Scientific Instruments, Kyoto, Japan), interfaced with Shimadzu gas chromatography (GC) apparatus. The GC was equipped with an Optima 5-MS fused silica capillary column (5% diphenyl - 95% dimethylpolysiloxane) (Macherey-Nagel, Düren, Germany) (length: 30 m; diameter: 0.25 mm; 0.25 μm film thickness), with helium as the carrier gas (1 ml/min), and programmed 5 min isothermal at 40 °C, then 40 °C to 150 °C at 4 °C/min, then to 240 °C at 16 °C/min. DEET mass spectrum was identified by matching the mass spectrum with data of Wiley registry of Mass Spectral Data (9th ed.), NIST MS Database (2011) and Adams software library (2007). DEET peak areas for each time point (T+0 to T+6h) were then compared to the mean DEET peak area of impregnated nets.

### Second blood-feeding and exposure to DEET

A second blood meal was provided to female mosquitoes 3 or 4 days after the first. Mosquitoes were starved for 1 day. For this blood meal, each group of mosquitoes (i.e. DEET pre-exposed and control) were split into two subgroups for the second exposure: half were exposed to ethanol and the other half to DEET. Therefore, after the first and second blood meal, we obtained the following four treatments according to the chemical mosquitoes were exposed: (i) DEET-DEET; (ii) DEET-ethanol; (iii) ethanol-DEET; and (iv) ethanol-ethanol. For each replicate during this second blood meal, a subset of DEET pre-exposed and control mosquitoes were followed individually during blood-feeding. To do this, female mosquitoes were individually placed into 30 ml tubes, whereas the remaining mosquitoes were placed into paper cups at 25 females per cup for a grouped blood meal, using the same device as described above for the first exposure. Individual and grouped females were provided the second blood meal again through DEET or ethanol impregnated nets and a parafilm membrane. The dose of 250 mg/m^2^ of DEET was used on 15 × 17 cm nets for the grouped mosquitoes and 7 × 7 cm for the individual mosquitoes. Feeders for grouped mosquitoes were filled with 500 μl of blood, whereas for individually-exposed females they were filled with 200 μl. After 1 h of exposure, the number of blood-fed females was counted, and they were all kept singly in the same conditions as during the rearing in order to monitor life-history traits. Size of the blood meal, oviposition rate, fecundity, fertility, emergence rate of the offspring and survival were used as fitness indicators to assess the long-term impact of DEET exposure on mosquitoes. Additionally, as the irritancy of DEET may affect the number of attempts to blood-feed and thus the energy spent to take a blood meal may increase, female activity during blood-feeding was also measured.

To control for a potential genetic selection of DEET insensitivity which could affect the feeding success under the presence of DEET, the offspring from the tested females were kept and the descendant females were exposed to a blood meal in the presence/absence of DEET according to the protocol used for the second blood meal of their mothers. Comparisons of the blood-feeding success through DEET-impregnated nets between offspring females from DEET-exposed mothers and from control mothers were thus carried out.

### Recording of life history traits

#### Activity monitoring

Flight activity during exposure to the second blood-feeding was monitored in individually-tested mosquitoes using a locomotor activity monitor system (TriKinetics, Waltham, MA, USA). The device consists of a series of infrared LEDs placed around a 30 ml tube where the mosquito is placed, and each time it crosses the infrared beams, these are interrupted. The infrared beams were placed next to the membrane feeder and as a mosquito came back and forth to the provided blood, interruption of the beams was recorded. The number of interruptions (i.e. crossings) was used as a proxy of the mosquito flight activity during the assay.

#### Blood meal size

The quantity of excreted haematin during blood digestion was used to assess the quantity of ingested hemoglobin during the first and second blood meals [29]. To do this, a subset of blood-fed females during the first blood meal and all fed females during the second blood meal were maintained in individual vials for 48 h. Female mosquitoes were then removed, and excreta was eluted in 1 ml of 1% lithium carbonate solution. Absorbance of the eluate was measured at 390 nm by a VICTOR Multilabel Plate Reader (PerkinElmer, Waltham, MA, USA). The quantity of excreted haematin by individual females was estimated by using the standard curve obtained from known concentrations of porcine haematin (1–100 μg/ml) (Sigma-Aldrich) and used as a proxy for the blood meal size.

#### Fecundity, fertility and survival

After ingestion of the first blood meal and digestion, groups of 30 fed females were placed into cages with moist cotton and filter paper for oviposition. After the second blood meal and digestion, all females were individually placed in paper cups. The bottom of the cup was covered with moist cotton and filter paper to allow oviposition. Eggs were collected on the moist cotton. Oviposition rate was measured in individual females, and the number of eggs laid by grouped or individual females was counted using Egg Counter [30] software. Eggs were then grouped by treatment and put into demineralized water. The number of adult descendants of each batch was counted to determine the number of offspring produced. The number of dead females was recorded every day to ascertain the survival.

### Statistical analysis

All statistical analyses were performed using the software R 3.3.2 [31].

#### Blood-feeding

Logistic regression by generalized linear mixed-effects model (*glmer*, binomial distribution, logit-link, *lme4* package [32]) was used to compare the proportion of blood-fed females after the first blood meal, with batch included as a random effect. The effects of exposure to DEET during the first and second blood meal on the blood-feeding success during the second exposure were also evaluated by *glmer* with binomial distribution. The nature of the first and second exposure was included as fixed effect, and replicate was coded as a random factor. *Post-hoc* comparisons between the four groups of treatment at the second exposure were performed using multiple comparisons (Tukey’s tests, *multcomp* package [33]). The effect of mother exposure on the success of blood-feeding of the offspring was analyzed by generalized linear model (*glm*, binomial distribution) with mothers and daughters exposure coded as a fixed factor.

### Life-history traits

In a first analysis, we tested for the effect of the first exposure on subsequent life history traits (type 2 ANOVA, *car* package [34]). Quantity of excreted haematin and fertility (mean number of descendants produced) were analyzed using a linear mixed-effects model with a Gaussian distribution, after sqrt-transformation and confirmation of data normality (*lmer* and *lme4* packages). Number of eggs laid (expressed as a mean per female) was also analyzed using *lmer* with a Gaussian distribution, without data transformation. Emergence rate (proportion of eggs that reached adult stage) was assessed by using *glmer* with binomial error distribution. For all these models, replicate was coded as a random factor.

In a second analysis, we tested for the effect of the first and the second exposure on each life history trait considered subsequent to the second exposure (type 2 and 3 ANOVA, *car* package). Quantity of excreted haematin, fertility and emergence rate were analyzed using the same methodology as described above. Oviposition rate was analyzed by *glmer* with binomial error distribution. Number of eggs laid subsequent to the second blood meal was obtained from individually-followed females and analyzed by generalized linear mixed models using AD Model Builder to account for over-dispersion (glmmadmb, negative binomial distribution, *glmmADMB* package [35]). Survival was evaluated with a mixed effects Cox proportional hazards regression model (packages *survival*, *coxme* [36, 37]). For these models, the nature of the first and the second exposure was included as a fixed factor and replicate was coded as a random factor. Flight activity was estimated by *glmmADMB* with the nature of the first and the second exposure coded as a fixed factor.

The influence of several explanatory variables was investigated by including in the models: mosquito age, replicates, and the type of exposure (grouped *vs* individual). The contribution of each explanatory variable was assessed sequentially using ANOVA function, with non-significant terms removed from the model. Model selection was performed using AIC and analysis of the residuals (plotresid, *RVAideMemoire* package [38]). Results are presented as mean ± standard error (SE) and proportion ± 95% confidence interval (CI).

## Results

### Biological relevance of the used concentration of DEET

**Fig. 1:**
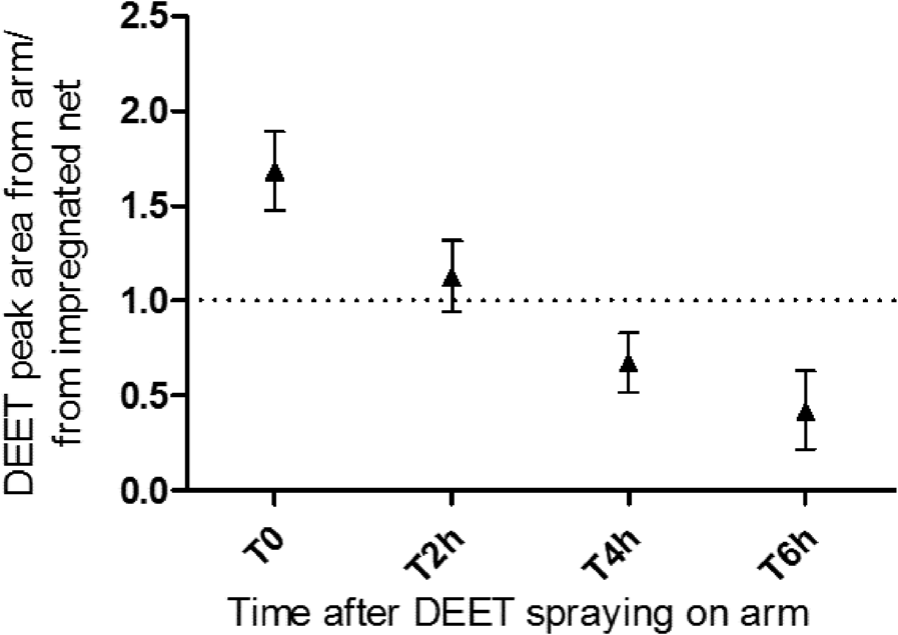
Ratios of the mean DEET gas chromatogram peak areas from forearms *versus* mosquito nets. Forearms were sprayed with DEET at 2 g/m^2^ and mosquito nets were impregnated with DEET at 250 mg/m^2^. Value 1 corresponds to similar mean peak areas between arms and mosquito nets. Error bars show standard error (SE)

When female mosquitoes were provided a first blood meal through a net impregnated with 250 mg/m² of DEET *versus* control, DEET induced a 60% inhibition of blood meal (*χ*^2^ = 51.42, *df* = 1, *P* < 0.0001) showing that the dose used significantly affected the feeding success. Emanations of such impregnated net were measured in regards to sprayed DEET on skin according to the guidance for the use of DEET. Odor captures from volunteers’ arms with added DEET showed that the concentration used in the assays approximately corresponded to emanations of an arm two hours after DEET cutaneous application at the recommended dose (Fig. 1).

### Effect of a prior blood meal in contact with DEET on the success of the second blood meal upon a DEET-treated net

**Fig. 2:**
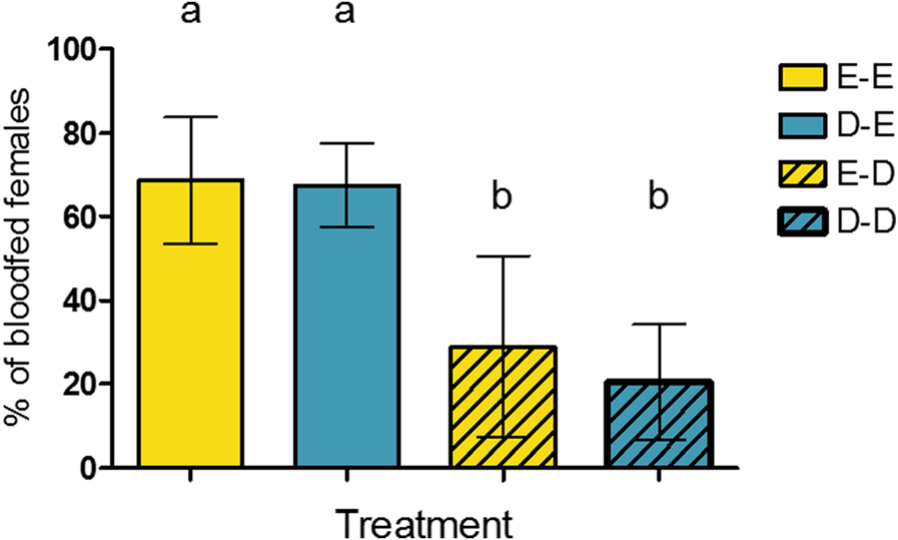
Proportion of blood-fed *An. gambiae* during the second blood meal for each treatment. Yellow bars represent females pre-exposed to ethanol (control), and blue bars represent females pre-exposed to DEET during the first blood meal. Full bars show females exposed to ethanol, hatched bars show females exposed to DEET during the second blood meal. Error bars show 95% confidence intervals (CI). Different letters indicate significant differences (*post-hoc* Chi-square tests with a Bonferroni correction, *P* < 0.05). *Abbreviations*: E, ethanol; D, DEET

Blood-feeding success at the second blood meal was tested for 706 females (219 for the grouped exposure and 487 for the individual exposure) across eight replicates. Generalized linear mixed model revealed no effect of the first exposure on the success of feeding during the second blood meal (*χ*^2^ = 0.18, *df* = 1, *P* = 0.67). The success of blood-feeding during the second blood meal was only influenced by the nature of the second exposure (*χ*^2^ = 105.42, *df* = 1, *P* < 0.0001), the presence of DEET inducing a 75% inhibition of the blood-feeding. When considering the four treatments at the second exposure (Fig. 2), paired comparisons showed no differences in the blood-feeding through an ethanol-treated net between DEET pre-exposed and control females (*P* = 0.99), as well as no differences in the proportion of blood-fed females upon a DEET-treated net between control and DEET-pre-exposed ones (*P* = 0.82). This confirms that the success at taking a blood meal at the second exposure is not influenced by the nature of the first exposure. Analysis on the offspring revealed no effect of mother exposure to DEET on the success of the daughters to blood-feed upon a DEET-treated net or an ethanol treated net (*χ*^2^ = 0.002, *df* = 1, *P* = 0.96).

### Effect of DEET exposure on mosquito life history traits after the first blood meal

**Fig. 3:**
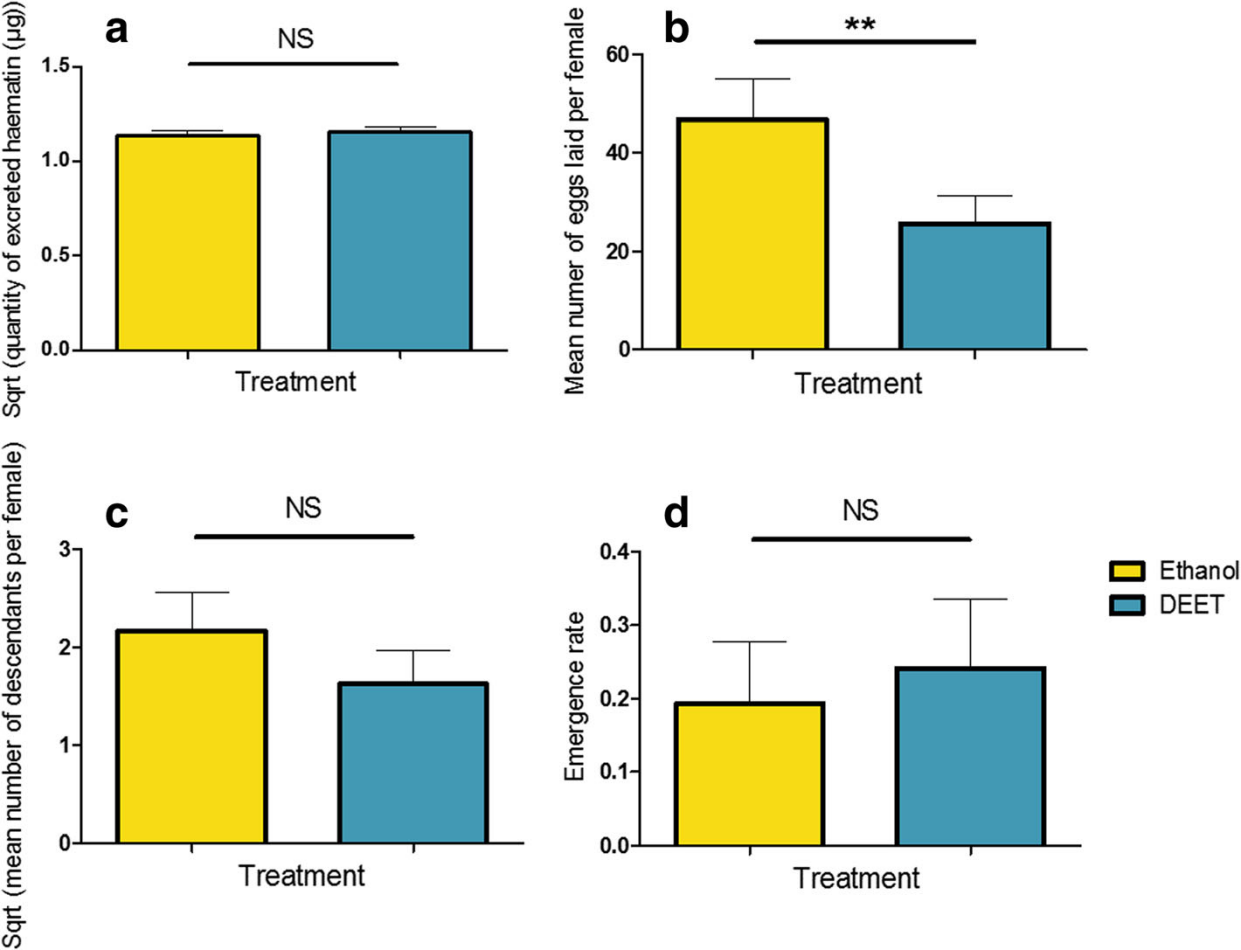
Effect of the first exposure to DEET on life history traits ensuing the first exposure: volume of blood ingested (**a**), fecundity (**b**), fertility (**c**) and offspring emergence rate (**d**). Yellow bars represent females pre-exposed to ethanol (control) and blue bars represent females pre-exposed to DEET. Results are presented as mean ± standard error (SE) (*post-hoc* Chi-square tests, *P < 0.05, **P < 0.01, ***P < 0.001)

The size of the blood meal was assessed using a subset of 173 blood-fed-females followed individually across 3 replicates. Fecundity, fertility and emergence rate were measured in the 1399 blood-fed females followed by group of 30 across eight replicates, and expressed as a mean per female. DEET exposure of female mosquitoes during the first blood meal revealed a strong impact on the subsequent number of eggs laid, with females pre-exposed to DEET laying significantly less eggs than control females (*χ*^2^ = 8.37, *df* = 1, *P* = 0.0038). DEET exposure at the first blood meal, however, did not affect the size of the blood meal (*F*_(1, 169)_ = 3.17, *P* = 0.077), the number of descendants produced (*χ*^2^ = 1.56, *df* = 1, *P* = 0.21) nor the emergence rate (*χ*^2^ = 1.67, *df* = 1, *P* = 0.20) (Fig. 3).

### Effect of DEET exposure on mosquito life history traits after the second blood meal

**Fig. 4:**
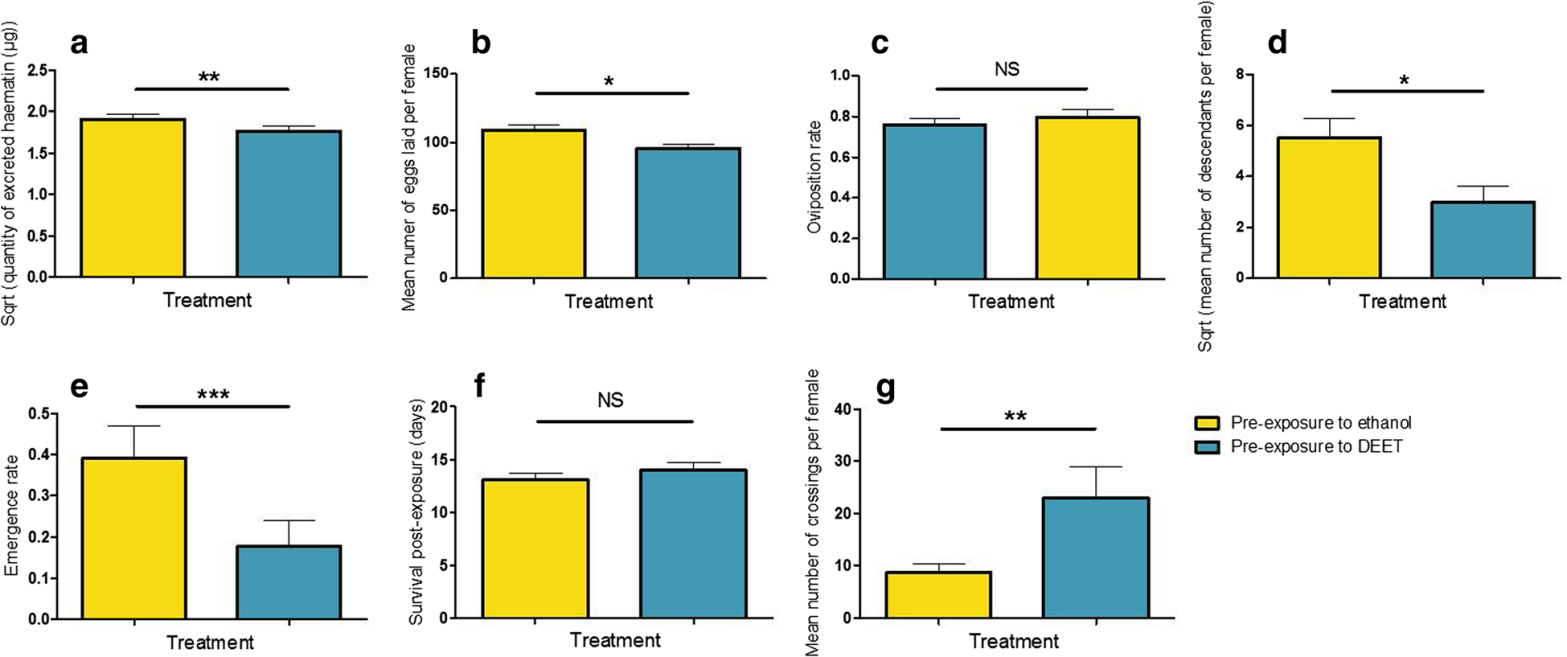
Effect of the first exposure to DEET on life history traits ensuing the second exposure: volume of blood ingested (**a**), fecundity (**b**), oviposition rate (**c**), fertility (**d**), offspring emergence rate (**e**), survival post-exposure (**f**) and activity (**g**). As the effect of the second exposure on the recorded life-history traits showed no significance, females were pooled considering the treatment they received at the first exposure, irrespective of the treatment they received at the second exposure. Yellow bars represent females pre-exposed to ethanol (control), and blue bars represent females pre-exposed to DEET. Results are presented as mean ± standard error (SE) (*post-hoc* Chi-square tests, *P < 0.05, **P < 0.01, ***P < 0.001)

Data related to life history traits after the second blood meal was collected in blood-fed females followed individually across eight replicates. Activity was assessed in a subset of 203 females during blood-feeding. The size of the blood meal, oviposition rate and survival were measured in 320 females. Fecundity was estimated in the 228 females that oviposited. For fertility and emergence rate measurement, larvae were grouped by treatment so data is expressed as a mean per female. A significant effect of the first exposure was observed on several life history traits ensuing the second blood meal (Fig. 4). Compared to control females, DEET-pre-exposed females showed a reduced volume of blood intake (*χ*^2^ = 9.034, *df* = 1, *P* = 0.0027), as well as a lower number of eggs laid (*χ*^2^ = 4.58, *df* = 1, *P* = 0.032) and a lower number of descendants produced (*χ*^2^ = 6.23, *df* = 1, *P* = 0.013). These effects were observed independently of the nature of the second exposure, as statistical models revealed no effect of the nature of the second exposure on these life history traits (*χ*^2^ = 0.58, *df* = 1, *P* = 0.45; *χ*^2^ = 0.0031, *df* = 1, *P* = 0.96 and *χ*^2^ = 0.065, *df* = 1, *P* = 0.80, respectively). Additionally, a significant interaction was observed between the two exposures regarding the emergence rate subsequent to the second blood meal. Yet, emergence rate is lower in the offspring of DEET-pre-exposed females (*χ*^2^ = 152.44, *df* = 1, *P* < 0.0001). Moreover, independently of the nature of the second exposure (*χ*^2^ = 1.30, *df* = 1, *P* = 0.25), mosquitoes that were pre-exposed to DEET showed a higher flight activity at the second exposure than control mosquitoes (*χ*^2^ = 8.02, *df* = 1, P = 0.0046). Additionally, oviposition rate and mosquito survival were neither influenced by the first (*χ*^2^ = 0.0001, *df* = 1, *P* = 0.99 and *χ*^2^ = 0.0017, *df* = 1, *P* = 0.97, respectively) nor the second exposure (*χ*^2^ = 0.63, *df* = 1, *P* = 0.43 and *χ*^2^ = 1.0045, *df* = 1, *P* = 0.32, respectively).

## Discussion

The present study offers new insights on the effects of multiple exposures to DEET repellent on a vector mosquito. Experiments showed that, in the tested conditions, a prior successful blood meal taken in spite of contact with DEET did not affect the success of blood-feeding at the subsequent exposure. These results contrast with previous observations on mosquitoes which suggested that a prior exposure to DEET reduces the aversion at the following exposure, and may thus affect repellent efficacy over time [19, 20]. These studies evidenced that behavioral repellency can be reduced both by conditioned learning (association of a reward and the repellent odor) [20] and by sensory habituation (olfactory receptors no longer respond to a stimulus after being stimulated) [19]. Our data thus do not support such mechanisms in the tested conditions. The discrepancy in these observations can be explained by differences in experimental setup, design and mosquito species. First, previous studies intended to evaluate mosquito learning abilities, and focused on olfactory repellency rather than contact repellency. In contrast, the present experiment aimed at mimicking blood-feeding through a DEET-impregnated net or on sprayed skin, so mosquitoes were exposed to a direct contact with the repellent during the blood meal. The exposure here thus involves olfactory and gustatory receptors, which contrasts with previous studies where only olfactory receptors were involved. Recently, progress in the understanding of DEET’s mode of action indicates that not only is olfaction implicated in DEET detection, but that DEET is also actively detected by gustatory receptors during contact [39]. Yet, little is known about the interactions between olfactory and gustatory stimulations during the learning process. Olfactory perception of DEET along with its irritancy to gustatory receptors during contact could have triggered more complex responses and prevented the processes of learning and memory. Additionally, because our objective was to test multiple exposures to DEET in epidemiologically relevant conditions, the time between the first and second exposure was chosen to correspond to a gonotrophic cycle; three or four days correspond to the natural period of time between blood meals, rather than the shorter times (a few hours to one day) used in previous studies. The time between the two exposures is expected to be a discriminant parameter that explains discrepancies with previous studies. Indeed, literature data showed that in both conditioned learning and sensory adaptation, insect behavior is impacted for a few minutes to 24 hours after exposure ([40–44], but see [45]). However, female blood-feeding behavior is inherent to gonotrophic cycles, and developing costly mechanisms such as associative learning must be compensated by the selective advantage they offer. This implies that, to confer selective value, learning abilities should persist at least between two gonotrophic cycles. For now, associative learning in mosquitoes in which the association was retained for several days has been evidenced only once to our knowledge [45]. This raises the consideration that mosquitoes must have the physiological faculties to be conditioned and to memorize an association between two stimuli over two gonotrophic cycles. However, it is possible that learning abilities identified in previous studies may persist beyond three days, even though experimental designs have not provided evidence for it. The absence of apparent long-term memory associated with the learning process could be the consequence of some parameters that were absent from our experimental design. Indeed, others stimuli may be determinant for the establishment of learning, such as host volatiles. Yet, some studies suggest that DEET may act by inhibiting the detection of host odors by mosquitoes [46–48]. As the present design aimed at evidencing the potential effects of DEET exposure using heat as a single attractant, it does not allow the observation of the potential combined effects of repellent and host volatiles on mosquito memory. However, DEET acts *via* a complex mode of action and was also shown to be efficient in the absence of attractant odors [49]. Moreover, the described behavioral changes following a prior exposure to DEET were observed both in the presence and absence of host volatiles [19, 20]. We do not exclude that, under an experimental design using host odors, contrasting effects of DEET exposure on mosquito behavior could be observed. It thus remains an open question whether DEET and host-specific cues can act synergistically in triggering a mosquito associative learning process.

From another perspective, the present study did not aim at testing for the effect of an operant conditioning in laboratory but rather for the effect of multiple exposures to repellents that mosquitoes may actually face over time in biological situations. As our experimental design consisted of one single exposure before testing, it is worth considering that learning requires a higher number of exposures and thus more gonotrophic cycles to take place. Finally, previous studies were performed using *Aedes aegypti*. The contrasted results observed could then lie in the choice of mosquito species, as *Aedes* and *Anopheles* could respond differently to the DEET treatment due to evolutive divergence. The fact that our study did not reveal an impact of a prior exposure to DEET on the success of blood-feeding at the subsequent exposure supports the efficacy of this repellent for protection against mosquito bites. However, further investigations to determine the actual effect of multiple exposures in diverse environmental conditions and mosquito species are still needed.

Interestingly, the present study showed that the contact with DEET during a blood meal affects mosquito life history traits. First, DEET-pre-exposed mosquitoes showed a reduction in both fertility and fecundity, suggesting an overall reduction in the number of offspring produced. These observations are consistent with literature showing decreases in blood engorgement levels in *Ae. aegypti* up to several hours after exposure to DEET [50]. As only the effect of the treatment during the first blood meal is significant and observed after the second blood meal, this DEET effect does not appear to be the consequence of additive effects of the two blood meals, but rather a delayed effect of the first exposure that is expressed beyond the second exposure, even though mosquitoes are no longer exposed to DEET. It can be hypothesized that, since DEET acts as a toxicant against mosquitoes, the energy needed for detoxifying may be used to the detriment of the energy needed for reproduction. This hypothesis needs further investigation, as a possible cost of the detoxification following repellent exposure has, to our knowledge, never been investigated. Then, although mosquito survival was not observed to be affected by DEET exposure in our experimental design, the overall reduction in the number of offspring produced for DEET exposed females may potentiate DEET efficacy to control mosquitoes.

Pre-exposure to DEET also induced an increase of the flight activity during the subsequent blood meal, independently of the nature of the second exposure. DEET acts as an excito-repellent that has been shown to inhibit cholinesterases, which directly affects insect activity [51–53]. Although our study did not find any effect of the increased activity on the insect survival, it can be hypothesized that multiple exposures to DEET may affect the mosquito nervous system in a way that may, in the long-term, impact their survival or their ability to find hosts, mates, or suitable oviposition sites.

Taken together, these results strengthen the idea that the development of repellent-based control methods could potentiate the existing strategies in the fight against vector-borne diseases. Long-lasting-repellent-treated-nets alone or in combination with insecticides could allow the restoration of the efficacy of impregnated nets against nocturnal insecticide-resistant mosquitoes. Indeed, the main weakness of repellents is their volatility, but it has recently been shown that they could be encapsulated for a slow diffusion and be efficient over a period of months [54, 55]. When used as skin aerosols, repellents such as DEET also have a substantial potential in fighting diurnal mosquitoes, and could also affect insecticide-resistant mosquitoes. Moreover, the use of repellents could allow a decrease in the amount of insecticides involved in vector control programmes. This will help slowing down the spread of resistance mechanisms and reducing the public health challenges caused by the use of insecticides, such as toxicity for non-target insects, persistence in the environment, and human and animal health issues. Additionally, although genetic insensitivity to DEET can be selected in laboratory [56], no evidence for repellent-resistance was found in nature. As repellents do not kill their target but rather deter it for its initial host, the selective pressure induced by the use of repellents may be expected to be lower compared to the one induced by the use of insecticides. Consequently, resistance mechanisms consecutive to the massive use of repellents are less prone to spread rapidly, making repellents potential evolutionary-proof active compounds available to fight pathogen transmission [57].

It is worth noting that a risk that should be taken into account for the establishment of new control strategies is that a reduction of the blood engorgement, such as we observed in DEET-pre-exposed mosquitoes, could have implications on mosquito vectorial capacity, as it has been associated with increased re-feeding [50, 58]. Further investigation is needed to determinate the impact of DEET exposure on mosquito subsequent feeding behavior in natural conditions.

## Conclusions

The present study indicates that, under our experimental design, a successful blood meal obtained upon a DEET-treated net does not affect the success of blood-feeding in spite of DEET at the subsequent exposure in *An. gambiae*. However, we evidence here for the first time deleterious effects of DEET exposure on mosquito life-history traits related to fecundity and fertility. The data gathered in this study highlight the potential of repellents such as DEET for controlling vector mosquitoes. Although the observed deleterious effects of DEET exposures on mosquitoes are not expected to significantly reduce transmission alone, they should be taken into account in predictions of efficacy of repellent-based interventions. Additionally, because the use of insecticides alone may no longer be sustainable, repellents could be part of integrated management programs based on complementary and alternating tools that likely avoid the appearance of resistance mechanisms.

## Abbreviations

DEET: N,N-diethyl-3-methylbenzamide
